# Support for Texting-Based Condom Negotiation Among Forcibly Displaced Adolescents in the Slums of Kampala, Uganda: Cross-sectional Validation of the Condom Use Negotiated Experiences Through Technology Scale

**DOI:** 10.2196/27792

**Published:** 2022-04-06

**Authors:** Moses Okumu, Carmen H Logie, David Ansong, Simon Mwima, Robert Hakiza, Peter A Newman

**Affiliations:** 1 School of Social Work, University of Illinois, Urbana-Champaign Urbana, IL United States; 2 School of Social Sciences, Uganda Christian University Mukono Uganda; 3 Factor Inwentash Faculty of Social Work, University of Toronto Toronto, ON Canada; 4 United Nations University Institute for Water, Environment, and Health Hamilton, ON Canada; 5 School of Social Work, University of North Carolina at Chapel Hill Chapel Hill, NC United States; 6 AIDS Control Program, Ministry of Health Kampala Uganda; 7 Bukedi Prevention Institute Kampala Uganda; 8 Young African Refugees for Integral Development Kampala Uganda

**Keywords:** condom negotiation, sexting, refugee and displaced adolescents, digital sexual communication, HIV prevention, gender

## Abstract

**Background:**

Promoting sexual health among forcibly displaced adolescents is a global public health priority. Digital sexual communication strategies (eg, sexting) may increase adolescents’ confidence in discussing sexual health issues and negotiating condom use. However, limited evidence exists describing validated measures for text-based condom negotiation in the literature.

**Objective:**

This study helps fill this gap by adapting and examining the psychometric properties of a condom use experience through technology (condom use negotiated experiences through technology [CuNET]) scale.

**Methods:**

Using peer network sampling, 242 forcibly displaced adolescents (aged 16-19 years) living in Kampala’s slums were recruited for participation between January and March 2018. A subscale (embarrassment to negotiate condom use) of the Multidimensional Condom Attitudes Scale was adapted to incorporate sexting, yielding CuNET. Participants were randomly assigned to calibration and validation subsamples to conduct exploratory and confirmatory factor analyses to establish and validate the scale. CuNET measured participants’ support levels for texting-based condom negotiation via sexting based on gender, and multivariable logistic regression was used to explore its associations with sexual health outcomes (recent consistent condom use, access to sexual and reproductive health services, and lifetime sexually transmitted infection testing).

**Results:**

The one-factor CuNET with the validation sample was valid (*χ*^2^_4_=5.3; *P*=.26; root mean square error of approximation=0.05, 90% CI 0.00-0.16; comparative fit index=0.99; Tucker-Lewis index=0.99; standardized root mean square residual=0.006), and reliability (Cronbach α=.98). Adolescent girls showed significantly lower levels of support for using sexting to negotiate condom use (mean 13.60, SE 0.70 vs mean 21.48, SE 1.23; *P*=.001). In multivariable analyses, a 1-point increase in the CuNET score was associated with increased odds of recent consistent condom use (adjusted odds ratio [aOR] 1.73, 95% CI 1.24-2.41) but not with access to sexual and reproductive health services (aOR 1.51, 95% CI 0.99-2.30) or lifetime sexually transmitted infection testing (aOR 0.90, 95% CI 0.64-1.26).

**Conclusions:**

The unidimensional CuNET scale is valid and reliable for forcibly displaced adolescents living in slums in Kampala, gender-sensitive, and relevant for predicting consistent condom use among urban displaced and refugee adolescents. Further development of this scale will enable a better understanding of how adolescents use digital tools for condom negotiation.

## Introduction

### Background

Promoting sexual health among forcibly displaced adolescents is a global public health priority. A 2019 systematic review of sexual health interventions in humanitarian settings highlighted the urgent need for sexual health interventions targeting adolescents [1]. Uganda hosts over 1 million forcibly displaced persons, over 72,000 of whom live in Kampala [2], with an HIV prevalence rate of more than twice (13.9%) [3] the national average (6.2%) [4]. In a 2019 cross-sectional study of youth (n=1134) living in Kampala’s slums, 9.9% of participants reported HIV or sexually transmitted infection (STI) co-infection [5]. Despite the heightened risk of HIV among adolescents, condom use remains sporadic [6]. In a sample of slum-dwelling youth in Kampala attending vocational training, Swahn et al [7] found that 1 in 3 participants did not use condoms, whereas 44.9% had sex with multiple partners and 23.2% engaged in transactional sex. To improve the sexual health of vulnerable adolescents, the World Health Organization [8] is calling on public health advocates to leverage digital technologies, as digital media use is widespread among adolescents.

At the same time, there is scant evidence of valid and reliable measures for evaluating the effectiveness of digital sexual health interventions. Despite preliminary studies investigating the effect of digital sexual communication on condom self-efficacy and use, there is limited evidence of validated measures to study this effect [9,10]. This knowledge gap impedes researchers’ understanding of why and how today’s adolescents are deploying digital sexual communication to negotiate for safer sex.

### Digital Sexual Communication and Safer Sex

Digital technologies are (1) integral socialization tools for adolescents, many of whom regularly use texting apps and phone-based social media apps [11], and (2) becoming increasingly recognized as useful sexual health delivery tools because of their low cost and the privacy they provide, especially for adolescents [12,13]. For instance, using baseline data from a longitudinal study of 284 US adolescents, Widman et al [14] found that many adolescents used technology to negotiate for safer sex practices such as condom use, HIV or STI testing, and limiting sexual partners. Moreover, sexually active adolescents are more likely to engage in *sexting* (ie, a digital sexual communication strategy that involves sending or receiving sexually explicit materials through their mobile technologies) [10] to promote condom use [15]. A recent cross-sectional study of forcibly displaced adolescents in Kampala found that adolescents who engaged in sexting reported condom use compared with those who did not sext [16]. Adolescents’ digital communication practices require validated measures for evaluation.

### Measuring Digital Sexual Communication: Condom Use Negotiated Experiences Through Technology

This study combined items from one subscale (ie, embarrassment about using and negotiating condom use) of Helweg-Larsen and Collins [17] Multidimensional Condom Attitudes Scale (MCAS) and sexting data to yield the condom use negotiated experiences through technology (CuNET) scale, and then tested the psychometric properties and utility of the CuNET scale. The MCAS is the most commonly used scale to measure individual beliefs about and attitudes toward condoms [17]. The MCAS comprises five subscales measuring respondents’ opinions regarding (1) the reliability and effectiveness of condoms, (2) sexual pleasure associated with condom use, (3) stigma attached to condom use, (4) embarrassment about the purchase of condoms, and (5) embarrassment about condom use and condom negotiation [17,18]. Studies have found that the fifth subscale, embarrassment surrounding condom use and negotiation, is the most important factor in predicting condom use [17,19]. Given this subscale’s predictive power, measures of condom negotiation should incorporate contextual (ie, cultural socialization) and individual (ie, knowledge of and confidence with condoms) attributes that could affect condom use.

Preventive interventions informed by Social Cognitive Theory (SCT) [20] have demonstrated effectiveness in increasing condom use confidence and reducing STI incidence by equipping individuals with negotiation skills and increasing their confidence and ability to use condoms effectively. Yet measures of condom use likelihood have not been updated considering the significant changes in young people’s (often technology-based) negotiations of sexual activity. For instance, digital technologies may have shifted young people’s perceptions of sexual risk practices and attitudes toward condoms [10,21,22]. Digital technologies may alter adolescents’ attitudes and embarrassment about negotiating condom use by providing a digital environment for these discussions. As sexual health scholars and preventionists evaluate how best to leverage these new digital environments for sexual health interventions [11], they will need valid and reliable measures that recognize these emergent digital environments.

### Gender Sensitivity

Valid and reliable measures or scales for future digital sexual health interventions must also acknowledge how gender affects attitudes toward sexual activity, condom use, and negotiation. Gendered socialization in many societies emphasizes the control and dominance of men, with different gender-based expectations (eg, concerning sex) assigned to men and women [23,24]. Even though adolescent girls may be using new digital spaces to challenge traditional cultural sexual scripts that cast them as passive actors and sexual gatekeepers for men, evidence shows that sexting scripts have many similarities to traditional scripts [25]. In 2017, a review of qualitative literature, including 8 studies mostly from the United States (age range 12-25 years), showed that adolescent girls were asked far more often than adolescent boys to send nude pictures [26]. Similarly, a cross-sectional study conducted among 1653 adolescents in Sweden found that 26.2% of girls aged between 15 and 16 years in romantic relationships sent sexts to their partners, compared with 19.6% of boys [27]. Although the study authors did not provide a rationale for these different sexting rates, they are indicative of power imbalances and cultural expectations that compel girls to be responsive to the sexual needs of boys rather than asserting their own sexual needs. Collectively, these findings emphasize that any scale developed to assess sexting practices and inform digital sexual health interventions must be gender sensitive.

### This Study

This study contributes to the current sexting, digital sexual communication, and sexual health literature by modifying and validating a gender-sensitive measure that evaluates adolescents’ attitudes about using sexting to negotiate for condom use. Drawing from sexting literature [9,10,28], we hypothesized that adolescents who engage in sexting might demonstrate higher rates of condom use, as indicated by their low levels of embarrassment in using sexting to negotiate condom use. Using our adapted CuNET scale (ie, low embarrassment to negotiate and discuss condom use over sexting), we compared support for condom negotiation via sexting across gender among a sample of forcibly displaced adolescents in Kampala, Uganda, and tested the relationship between the CuNET scale and sexual health outcomes.

## Methods

### Participant Recruitment

From January to March 2018, in collaboration with 3 refugee-serving organizations and 2 government agencies, 242 forcibly displaced adolescents were recruited to participate in the study. Eligible participants ranged from the ages 16 to 19 years and (1) self-identified as a refugee or displaced person or having refugee or displaced parents, (2) reported that they lived in 1 of 5 slums settlements (Kabalagala, Rubaga, Kansanga, Katwe, and Nsambya), and (3) were able to provide informed consent. A total of 12 peer research assistants (PRAs), who self-identified as refugees or displaced persons aged 18 to 24 years, were also recruited and trained in research methods and confidentiality to help with participant recruitment and survey administration.

### Ethics Approval

The University of Toronto (#35405) and the Uganda Ministry of Health (ADM: 105/261/01) granted ethical approval for the research. Before completing the surveys, all participants provided written informed consent. Following guidance and waivers from the ethics boards, participants aged between 16 and 17 years were deemed capable of providing informed consent.

### Sampling and Data Collection

A peer-networking technique [29], an effective strategy to recruit and include marginalized populations, such as refugees and displaced youth, was used to recruit adolescents. Participants received recruitment vouchers from PRAs to help recruit 2 to 5 other forcibly displaced adolescents in their social network until the target number of participants was reached. Participants chose a private space for the PRA to administer the 35- to 45-minute structured survey on tablets in English or Swahili. Sexual health toolkits, counseling services, and a transport refund of USh 12,500 (approximately US $ 3.74) were provided to all participants who completed the survey.

### Measures

The MCAS’s embarrassment about negotiation and condom use subscale [17]—a 5-item scale that uses a Likert-type scale ranging from *strongly disagree*=1 to *strongly agree*=7; Cronbach α=.83—has been identified as the strongest predictor of condom use among all MCAS subscales. Therefore, this subscale was selected for adaptation to include sexting. As part of the adaptation process, feedback was solicited from practitioners, PRAs, and experts in Uganda before pilot testing the updated instrument among PRAs (n=12) and forcibly displaced adolescents (n=4) in Nsambya. Participants read the instructions and each item aloud, commented in their own words on what they were being instructed to do and what each item brought to their mind, picked a response option, and explained why they chose the option. After pilot testing the instrument on a number of people, the researchers conducted a debriefing session with participants to look for patterns in the feedback. Specifically, the researchers wanted to know whether participants had similar hesitations, requests for clarification, and whether they had any suggestions for different wording. Feedback during the pilot study suggested that statements should be reverse worded to avoid the response set. For instance, “When I suggest using a condom, I am almost always embarrassed” became “While sexting, I am not embarrassed to suggest using condoms to my partner” (Textbox 1). This recommendation is supported by Boateng et al [30], who argue that negatively worded or reverse-scored items have the potential to negatively impact a scale’s psychometric properties.

Participants in the pilot test also recommended that detailed response anchors be provided for clarity. The instrument was revised to include detailed responses with corresponding numbers: *strongly disagree*=1, *disagree*=2, *somewhat disagree*=3, *neither agree nor disagree*=4, *somewhat agree*=5, *agree*=6, and *strongly agree*=7. After the participants approved the final revised measure, it was named CuNET. The CuNET scale response items include, “While sexting, it is really easy to bring up issues of using condoms to my partners,” and “While sexting, I know what to say to my partner when I want to talk about condoms or other protections.” Textbox 1 shows the final version of the CuNET scale used in this study.

**Condom use negotiated experiences through technology observed indicators adapted for a cross-sectional sample of forcibly displaced adolescents in the slums of Kampala, Uganda.**

**Original items from Multidimensional Condom Attitudes Scale**

- When I suggest using a condom, I am almost always embarrassed
- It is really hard to bring up the issue of using condoms to my partner
- It is easy to suggest to my partner that we use a condom
- I’m comfortable talking about condoms with my partner
- I never know what to say when my partner and I need to talk about condoms or other protection

**Items used in this study**

- While sexting, I am not embarrassed to suggest using condoms to my partner
- While sexting, it is really easy to bring up issues of using condoms to my partner
- While sexting, it is easy to suggest to my partner that we use a condom
- While sexting, I am comfortable talking about condoms with my partner
- While sexting, I know what to say to my partner when I want to talk about condoms or other protections

To assess validity, associations between CuNET scores and sexual health outcomes (ie, access to sexual and reproductive health (SRH) services, lifetime STI testing, and recent consistent condom use) were examined among participants who were sexually active. Access to SRH was measured using a single self-reported item asking whether participants had ever accessed SRH services in the previous 3 months. Lifetime STI testing was assessed using a single item asking participants whether they had ever received an STI test. Recent consistent condom use was assessed using a single item asking participants whether they consistently used condoms in the previous 3 months. For all 3 items, the responses included yes=1 and no=0.

### Statistical Analyses

Descriptive, bivariate analyses, multivariable regression and exploratory factor analysis (EFA) were conducted using Stata 14 (StataCorp), whereas confirmatory factor analysis (CFA) was conducted using Mplus 7.4 [31]. Missing data were less than 5% that any procedure used for handling missing data would have resulted in similar results. First, descriptive analyses of all the variables were conducted. The steps were followed as outlined in Bowen and Guo [32] for testing measurement models by first creating two subsamples using a random process: calibration and validation. Specifically, Bowen and Guo recommend four steps for establishing a measure’s validity and reliability: (step 1) using EFA and calibration sampling to test the factor structure of the data, (step 2) conducting CFA and calibration sampling to test for construct validity of the scale, (step 3) using the full sample, chi-square independence test and 2-tailed independent *t* tests to conduct a gender sensitivity analysis of individual items and the entire scale, respectively, and (step 4) using the full sample and multivariable logistic regression to examine the predictive validity of the scale. The following steps allow a comprehensive understanding of the validity and reliability of the scale.

### Factor Structure

In step 1, the calibration subsample (N=121) was used to conduct an EFA to classify individual items into CuNET factors. Factor loadings that were ≥0.30 were retained [33]. The number of factors was determined by identifying eigenvalues >1 (Kaiser criterion) and via visual examination of the scree plot [34]. To test whether the sample was sufficient for conducting factor analysis, the Kaiser-Meyer-Olkin test (score above 0.60 recommended) and Bartlett test of sphericity (acceptable if statistically significant) were used [33].

### Construct Validity

Following the EFA, CFA was conducted in step 2 using the validation subsample (N=121) to verify the one-factor structure from the EFA results. As the CuNET response set was ordinal (ie, 7 categories), weighted least square mean and variance adjusted estimators were used for the CFA because weighted least square mean and variance adjusted estimators provide a more accurate parameter estimate and a model fit that is more robust to ordinal data [31]. Model fit was assessed using (1) comparative fit index (CFI), with adequate fit represented as CFI≥0.90; (2) standardized root mean square residual (SRMR), acceptable if ≤0.08; and (c) the root mean square error of approximation (RMSEA), acceptable if ≤0.08 [35,36]. A good model fit is indicated when CFI≥0.95, RMSEA≤0.05, and SRMR≤0.06 [36].

### Reliability

Calibration and validation data were combined, and items were summed up before assessing the reliability of the CuNET scale. Reliability was evaluated using Cronbach α, with values higher than .70 deemed acceptable [37]. Given that all items in the CuNET scale start with the words “While sexting,” Cronbach α may be inflated because of similarities in some of the wordings of the scale. Therefore, reliability was further evaluated using the composite reliability index, with a value of or above 0.70 deemed acceptable [33]. In addition, convergent validity was assessed using the average variance extracted (AVE), with a value above 0.50 deemed acceptable [33].

### Gender Sensitivity

In step 3, after confirming the one-factor CuNET scale, the full sample (combining the calibration and validation data) was used to examine gender differences. Here, a higher score indicated positive support for texting-based condom negotiation. Independent *t* tests were then used to compare the mean scores of the CuNET scale by gender.

The next aim was to analyze gender differences among participants who reported positive versus negative support for texting-based condom negotiation. After validating the CuNET scale, a dichotomous variable was created to categorize participants as *positive supporters* or *negative supporters*. Positive supporters (coded as 1) included participants who selected the response anchors *somewhat agree*=5, *agree*=6, and *strongly agree*=7. Negative supporters (coded as 0) included participants who selected the response anchors *strongly disagree*=1, *disagree*=2, *somewhat disagree*=3, and *neither agree or disagree*=4. A chi-square independence test was then conducted.

### Predictive Validity

In step 4, using the full sample, we conducted multivariable logistic regression to examine associations between CuNET and sexual health outcomes of recent consistent condom use, access to SRH services, and lifetime STI testing, adjusting for sociodemographic factors (eg, age, gender, education, and mobile phone use) factors. For this analysis, we tested 3 models among a sample of only adolescents who indicated to have engaged in sexual intercourse. We also calculated adjusted odds ratios (aORs), highlighting those significant at the *P*<.05 level. Missing responses were excluded from the analyses; the number of complete responses was reported for each variable.

## Results

### Sample Characteristics

As illustrated in Table 1, the average age of the participants was 17.56 (SD 1.10) years. More than 3 in 4 (196/242, 81%) were identified as adolescent girls, 77.8% (179/230) had less than secondary/ secondary education, 61.2% (148/242) were originally from the Democratic Republic of the Congo, and 63.2% (153/242) had been in a dating relationship in the last 12 months. About 61.6% (149/242) owned and used mobile phones, whereas over half (122/242, 50.5%) used more than one type of mobile app. Approximately 15.3% (37/242) engaged in digital sexting and 44.2% (107/242) self-reported having had sexual intercourse in their lifetime.

**Table 1:** Characteristics of forcibly displaced adolescents in the slums of Kampala, Uganda: cross-sectional findings (N=242).

Variables			Values
**Sociodemographic factors**			
	Age (years), mean (SD; range)		17.56 (1.10; 16-19)
	**Gender, n (%)**		
		Girls	196 (81)
		Boys	46 (19)
	**Education (n=230), n (%)**		
		Secondary or less than secondary school	179 (77.8)
		Postsecondary education	51 (22.2)
	**Place of birth (n=242), n (%)**		
		South Sudan	22 (9.1)
		Burundi	50 (20.7)
		Democratic Republic of the Congo	148 (61.2)
		Rwanda	9 (3.7)
		Others	13 (5.4)
	**Time in Uganda (years; n=242), n (%)**		
		<1	16 (6.6)
		1-5	137 (56.8)
		>5	88 (36.5)
	**Immigration status (n=242), n (%)**		
		Refugees	216 (90)
		Seeking asylum or undocumented	24 (10)
	**Dating relationship, n (%)**		
		Not dating	88 (36.5)
		Dating	153 (63.2)
**Technology use**			
	**Mobile phone ownership and use (n=242), n (%)**		
		No	93 (38.4)
		Yes	149 (61.6)
	Average text messages sent per day		2.90 (1.75)
	**Mobile App use (n=241), n (%)**		
		No app	69 (28.5)
		1 type of app	51 (21.1)
		2-3 types of apps	89 (36.8)
		4 or more types of apps	33 (13.6)
**Digital sexual communication, n (%)**			
	**Sexting patterns (n=242)**		
		Nonsexters	205 (84.7)
		Sexters	37 (15.3)
**Sexual health, n (%)**			
	**Ever had sex (n=241)**		
		No	134 (55.6)
		Yes	107 (44.4)
	**Recent consistent condom use (n=100)**		
		No	74 (74)
		Yes	26 (26)
	**Access to SRH^a^ services (n=100)**		
		No	71 (71)
		Yes	29 (29)
	**Lifetime STI^b^ testing (n=100)**		
		No	93 (93)
		Yes	7 (7)

^a^SRH: sexual and reproductive health.

^b^STI: sexually transmitted infection.

### EFA Results

An EFA with 5 items and oblique rotation revealed a single-factor solution of the CuNET construct using the calibration sample (Table 2). The Kaiser-Meyer-Olkin measure of sampling adequacy was 0.89, exceeding the commonly recommended value of 0.60, and Bartlett test of sphericity was significant: *χ*^2^_10_=3418.9 (*P<*.001), eigenvalue=4.48, and communalities above 0.30.

**Table 2:** Summary of exploratory factor analysis results for CuNET^a^ scale using a calibration sample of forcibly displaced adolescents living in the slums of Kampala, Uganda: cross-sectional findings (N=121).

Item	Factor loading	KMO^b^
CuNET^b^1: While sexting, I am not embarrassed to suggest using condoms to my partner	0.91	0.93
CuNET2: While sexting, it is really easy to bring up issues of using condoms to my partner	0.96	0.93
CuNET3: While sexting, it is easy to suggest to my partner that we use a condom	0.97	0.85
CuNET4: While sexting, I am comfortable talking about condoms with my partner	0.96	0.83
CuNET5: While sexting, I know what to say to my partner when I want to talk about condoms or other protections	0.94	0.92
KMO	N/A^c^	0.89

^a^CuNET: condom use negotiated experiences through technology.

^b^KMO: Kaiser-Meyer-Olkin measure of sample adequacy.

^c^N/A: not applicable.

### CFA Results

The one-factor CuNET with the validation sample showed good fit: *χ*^2^_4_=5.3 (*P=*.26), RMSEA=0.05 (90% CI 0.00-0.16), CFI=0.99, Tucker–Lewis index=0.99, and SRMR=0.006 (Tables 3 and 4).

Factor loadings ranging from 0.91 to 0.98 exceeded the recommended 0.30 cutoff for the modified instruments. Evidence from the CFA using the validation subsample provides statistical support to the unidimensional model of the CuNET construct in the sample.

**Table 3:** Covariance matrix of the confirmatory factor analysis for the CuNET^a^ scale using a validation sample of forcibly displaced adolescents living in the slums of Kampala, Uganda: cross-sectional findings (n=121).

	CuNET 1	CuNET 2	CuNET 3	CuNET 4	CuNET 5
CuNET 1	4.07	—^b^	—	—	—
CuNET 2	3.86	4.58	—	—	—
CuNET 3	3.6	4.17	4.28	—	—
CuNET 4	3.67	4.05	4.02	4.36	—
CuNET 5	3.69	4.17	3.92	3.91	4.45

^a^CuNET: condom use negotiated experiences through technology.

^b^Not applicable.

**Table 4:** Confirmatory factor analysis for the CuNET^a^ scale using a cross-sectional validation sample of forcibly displaced adolescents living in the slums of Kampala, Uganda (n=121).

CuNET scale items		Factor loadings
CuNet 1: While sexting, I am not embarrassed to suggest using condoms to my partner		0.91
CuNet 2: While sexting, it is really easy to bring up issues of using condoms to my partner		0.98
CuNet 3: While sexting, it is easy to suggest to my partner that we use a condom		0.96
CuNet 4: While sexting, I am comfortable talking about condoms with my partner		0.96
CuNet 5: While sexting, I know what to say to my partner when I want to talk about condoms or other protections		0.93
**Fit indices from CFA^b^; one latent factor**		
	Chi-square	5.29^c^
	RMSEA^d^	0.05
	CFI^e^	0.99
	TLI^f^	0.99
**Internal consistency of the final model**		
	Number of items	5
	Cronbach α	0.98
	Average variance extracted	0.90
	Composite reliability index	0.98

^a^CuNET: condom use negotiated experiences through the technology scale.

^b^CFA: confirmatory factor analysis.

^c^*P=*.26.

^d^RMSEA: root mean square error approximation.

^e^CFI: comparative fit index.

^f^TLI: Tucker–Lewis index.

### Internal Reliability Results

Using the full sample, the values for Cronbach α (.98) and composite reliability index (0.98) exceeded the 0.70 thresholds, indicating that the CuNET scale had high internal consistency and reliability (Table 4). The results also provided evidence of convergent validity (AVE=0.90), as the CuNET scale exceeded the AVE threshold of 0.50.

### Support for Texting-Based Condom Negotiation

Table 5 presents levels of support for texting-based condom negotiation. Overall, approximately 1 in 4 respondents reported support for using sexting to negotiate for condom use.

Using independent *t* tests, the gender-based analysis showed that the combined CuNET scale was gender-sensitive: adolescent girls showed significantly lower levels of support for using sexting to negotiate condom use compared with adolescent boys (mean 13.60; SE 0.70 vs 21.48; SE 1.23; *P*<.001). Using CuNET scale item analysis, the chi-square test of independence revealed that adolescent boys showed significantly higher levels of support for using sexting to negotiate condom use in all 5 items. For instance, 61% (28/46) of adolescent boys agreed that “While sexting, I know what to say to my partner when I want to talk about condoms or other protections,” compared with 21% (41/196) of adolescent girls. Adolescent girls reported the lowest percentage (34/196, 17.4%) of support for the item asking if sexting could reduce their discomfort with suggesting condom use to their partners, compared with adolescent boys (21/46, 46%).

**Table 5:** Levels of CuNET^a^ among a cross-sectional sample of forcibly displaced adolescents living in the slums of Kampala, stratified by gender (5 items; N=242)^b^.

Items	Statements	Girls (n=196), n (% agreed)	Boys (n=46), n (% agreed)	Total, n (% agreed)	*P* value
CuNet 1	While sexting, I am not embarrassed to suggest using condoms to my partner	34 (17.4)	21 (45.7)	55 (22.8)	<.001
CuNet 2	While sexting, it is really easy to bring up issues of using condoms to my partner	38 (19.5)	24 (52.2)	62 (25.7)	<.001
CuNet 3	While sexting, it is easy to suggest to my partner that we use a condom	39 (20)	25 (54.3)	64 (26.6)	<.001
CuNet 4	While sexting, I am comfortable talking about condoms with my partner	43 (22.1)	26 (56.5)	69 (28.6)	<.001
CuNet 5	While sexting, I know what to say to my partner when I want to talk about condoms or other protections	41 (21)	28 (60.9)	69 (28.6)	<.001

^a^CuNET: condom use negotiated experiences through the technology scale.

^b^A chi-square independence test was conducted to examine how CuNET items differed by gender; agreed percentages were calculated by creating a categorical variable using a cutoff of 5 and above, which indicated positive support. The total is the percentage of participants who provided positive attitudes toward using sexing for condom negotiation.

### Associations Between Support for Texting-Based Condom Negotiation and Sexual Health Outcomes

In adjusted analyses, a 1-point increase in the CuNET score was associated with higher odds of recent consistent condom use versus no recent consistent condom use (aOR 1.73, 95% CI 1.24-2.41). Holding other factors constant, we found no association between CuNET and access to SRH services versus no access to SRH services (aOR 1.51, 95% CI 0.99-2.30) or recent lifetime STI testing versus never lifetime STI testing (aOR 0.90, 95% CI 0.64-1.26; Table 6).

**Table 6:** Independent association between CuNET^a^ and sexual health factors among a cross-sectional sample of sexually active forcibly displaced adolescents living in the slums of Kampala (N=100)^b^.

			Values		Recent consistent condom use				Values		Access to SRH^c^ services				Values			STI^d^ testing (Ever)		
					aOR^e^ (95% CI)		*P* value				aOR (95% CI)		*P* value				aOR (95% CI)			*P* value
CuNet, mean (SD)			24.31 (8.46)		1.73 (1.24-2.41)		*.001* ^f^		21.13 (8.60)		1.51 (0.99-2.30)		.05		14.31 (11.14)		0.90 (0.64-1.26)			.54
Age (years), mean (SD)			17.96 (0.96)		0.92 (0.33-4.05)		.84		17.60 (1.07)		0.26 (0.09-0.78)		*.02*		18.34 (0.97)		2.49 (1.19-5.22)			*.02*
**Gender (ref boys), n (%)**																				
	Girls	21 (80.8)		0.83 (0.19-3.67)		.81		19 (73.1)		0.79 (0.08-8.14)		.85		48 (96)		0.09 (0.01-0.89)			*.04*	
	Boys	5 (19.2)		N/A^g^		N/A		7 (26.9)		N/A		N/A		2 (4)		N/A			N/A	
**Mobile phone ownership (ref no mobile phone), n (%)**																				
	Yes	24 (92.3)		0.23 (0.04-1.51)		.13		12 (80)		0.37 (0.04-3.51)		.39		27 (84.4)		0.51 (0.51-12.36)			.26	
	No	2 (8.7)		N/A		N/A		3 (20)		N/A		N/A		5 (15.6)		N/A			N/A	
**Dating relationship (ref no dating relation), n (%)**																				
	Dating	26 (100)		1.15 (0.33-2.41)		.83		N/A		N/A		N/A		30 (93.8)		0.28 (0.07-1.16)			.08	
	Not dating	0 (0)		N/A		N/A		N/A		N/A		N/A		2 (6.3)		N/A			N/A	
**Education (ref postsecondary education), n (%)**																				
	Secondary or less than secondary school	12 (48)		0.74 (0.16-3.42)		.70		N/A		N/A		N/A		23 (71.9)		0.64 (0.12-3.43)			.61	
	Postsecondary education	13 (52)		N/A		N/A		N/A		N/A		N/A		9 (28.1)		N/A			N/A	

^a^CuNET: condom use negotiated experiences through technology.

^b^CuNET scale summarized scores were calculated for the 5 items; higher scores indicated higher support for using sexing to negotiate condom use. Due to distribution in the access to sexual and reproductive health services variable, only adjusted for 3 variables.

^c^SRH: sexual and reproductive health.

^d^STI: sexually transmitted infection.

^e^aOR: adjusted odds ratio.

^f^Statistically significant.

^g^N/A: not applicable.

## Discussion

### Principal Findings

Accurate measurement of forcibly displaced adolescents’ support for condom negotiation via digital tools may be important for the development and evaluation of digital sexual health interventions that aim to promote condom use. The findings from this study show that the one-factor CuNET scale is (1) valid and reliable for forcibly displaced adolescents living in informal settlements in Kampala, Uganda, (2) gender sensitivity, and (3) a portable tool for identifying factors associated with recent condom use among urban forcibly displaced adolescents.

### Comparison With Prior Work

The findings of this study depart from sexting literature that focuses solely on the harmful effects of technology on sexuality [15,21,22] by highlighting the positive applications that technology can have among forcibly displaced adolescents. Among adolescents who may be socialized to view conversations surrounding sex as taboo [23], sexting may provide them with convenient ways to communicate with each other privately. Indeed, a recent meta-analysis of 23 studies concluded that sexting was part of adolescents’ development and sexual exploration [10]. This study extends the measurement of condom negotiation strategies from face-to-face interactions to include interactions in digital environments and updates the MCAS—embarrassment to negotiate the condom use subscale [17] by recognizing sexting as a condom negotiation strategy. That is, sexting can be an important method by which adolescents influence their partners to use condoms, and thus a potentially high-impact target of sexual health interventions. As this study only focused on the utility of participants’ embarrassment to negotiate condom use via sexting, there is a need for future studies that develop a more comprehensive condom negotiation scale that encompasses other digital sexual communication strategies.

A closer look at the CuNET scale items indicates that confidence, knowledge, and persuasion are the key traits measured by the scale. These traits align with an important tenet of SCT—that for an individual to negotiate for condom use, they must have enough knowledge about the subject and confidence in their ability to engage their partner in conversations about sex-related topics. Importantly, this study shows that the venue of communication (ie, digital or in-person) may influence confidence levels surrounding these conversations. Digital technologies may afford adolescents with a continuous, digital, and real time venues to negotiate condom use with less embarrassment. Embarrassment may be addressed through the development and evaluation of interventions that encourage the exploration of sexual health subjects through technological interfaces.

Another primary goal of this study was to examine the gender sensitivity of the CuNET scale. The findings highlight gender differences in the endorsement of the CuNET scale items. Adolescent boys viewed the use of sexting for condom negotiation more favorably than adolescent girls. This finding is consistent with the sexual script theory [23], which highlights the double standards in sexual expectations for girls and boys. Adolescent girls may be socialized to avoid initiating any discussions of sex-related topics, regardless of venue. It is plausible that girls may support negotiating condom use via digital tools; however, they may not be willing to disclose this to peer interviewers because of the negative judgments attached to them doing so [38].

Findings from the psychometrics testing of the CuNET scale highlight how the changing landscape of digital sexual communications may provide an important opportunity to revisit condom negotiation strategies and interventions. Consistent with Widman et al [14], we found that favorable support for texting-based condom negotiation was associated with condom use. Thus, although texting-based condom negotiation may provide creative approaches that sexually active adolescents can use to negotiate condom use with their partners, interventions that increase condom education and access are critical. Therefore, interventionists need to consider gender differences and sociocultural norms that influence condom use and condom negotiation among adolescents.

### Limitations

The findings of this study should be interpreted within the context of its limitations. This study relies on data from a nonprobable sample of forcibly displaced adolescents living in slums of Kampala, Uganda. This sample may not necessarily be representative of forcibly displaced communities in Kampala, Uganda, and does not capture the diversity of the adolescent refugee population globally. Our study had more adolescent girls than adolescent boys, which limits our ability to compare the 2 genders. Originally, the study was designed to include only adolescent girls because of the high HIV rates, which was 3-fold than that of adolescent boys in Uganda. However, our local collaborators asked us to also include adolescent boys. As a result, we oversampled the proportion of girls to reflect the local HIV epidemic characteristics. The use of a cross-sectional design means that the causality of the predictive validity of the CuNET scale cannot be inferred. Future longitudinal studies are needed to explore whether CuNET scale scores predict later condom use. The inability to test group invariance because of the small sample size calls for future studies that use rigorous methods to validate the CuNET scale. Furthermore, although the scale was pilot-tested and modified according to participant feedback on its face and content validity, there is a need for future studies that draw together adolescent participants and adolescent health experts to develop a culturally responsive CuNET scale that can capture multiple possible ways in which adolescents engage in condom negotiation. In addition, the high reliability and internal consistency of the CuNET scale indicate that some items may have a close meaning. Future studies should further refine the CuNET scale to reduce redundancy in scale items.

### Conclusions

Despite these limitations, the strengths of this study include the validity and reliability of a new gender-sensitive scale (CuNET) useful for measuring support for texting-based condom negotiation among marginalized adolescents. The CuNET scale can be used to collect more targeted data for prevention studies addressing adolescents’ support for negotiating condom use via digital tools. The fact that CuNET is a digital sexual communication instrument responds to recent calls to leverage digital tools for the sexual health of adolescents [11,12]. On the basis of this study’s findings, more research is needed to develop further and validate a gender-responsive CuNET scale for adolescents. The current iteration of the CuNET scale appears to be a valid measure for assessing support for texting-based condom negotiation among forcibly displaced adolescents living in HIV hyperendemic settings in Kampala, Uganda, with strong potential for future adaptation to a host of settings and populations.

## Abbreviations

aOR: adjusted odds ratio
AVE: average variance extracted
CFA: confirmatory factor analysis
CFI: comparative fit index
CuNET: condom use negotiated experiences through technology
EFA: exploratory factor analysis
MCAS: Multidimensional Condom Attitudes Scale
PRA: peer research assistant
RMSEA: root mean square error of approximation
SCT: Social Cognitive Theory
SRH: sexual and reproductive health
SRMR: standardized root mean square residual
STI: sexually transmitted infection
